# Evidence of Authentic DNA from Danish Viking Age Skeletons Untouched by Humans for 1,000 Years

**DOI:** 10.1371/journal.pone.0002214

**Published:** 2008-05-28

**Authors:** Linea Melchior, Toomas Kivisild, Niels Lynnerup, Jørgen Dissing

**Affiliations:** 1 Research Laboratory, Institute of Forensic Medicine, University of Copenhagen, Copenhagen, Denmark; 2 Leverhulme Center for Human Evolutionary Studies, The Henry Wellcome Building, University of Cambridge, Cambridge, United Kingdom; 3 Laboratory of Biological Anthropology, Institute of Forensic Medicine, University of Copenhagen, Copenhagen, Denmark; Centre for DNA Fingerprinting and Diagnostics, India

## Abstract

**Background:**

Given the relative abundance of modern human DNA and the inherent impossibility for incontestable proof of authenticity, results obtained on ancient human DNA have often been questioned. The widely accepted rules regarding ancient DNA work mainly affect laboratory procedures, however, pre-laboratory contamination occurring during excavation and archaeological-/anthropological handling of human remains as well as rapid degradation of authentic DNA after excavation are major obstacles.

**Methodology/Principal Findings:**

We avoided some of these obstacles by analyzing DNA from ten Viking Age subjects that at the time of sampling were untouched by humans for 1,000 years. We removed teeth from the subjects prior to handling by archaeologists and anthropologists using protective equipment. An additional tooth was removed after standard archaeological and anthropological handling. All pre-PCR work was carried out in a “clean- laboratory” dedicated solely to ancient DNA work. Mitochondrial DNA was extracted and overlapping fragments spanning the HVR-1 region as well as diagnostic sites in the coding region were PCR amplified, cloned and sequenced. Consistent results were obtained with the “unhandled” teeth and there was no indication of contamination, while the latter was the case with half of the “handled” teeth. The results allowed the unequivocal assignment of a specific haplotype to each of the subjects, all haplotypes being compatible in their character states with a phylogenetic tree drawn from present day European populations. Several of the haplotypes are either infrequent or have not been observed in modern Scandinavians. The observation of haplogroup I in the present study (<2% in modern Scandinavians) supports our previous findings of a pronounced frequency of this haplogroup in Viking and Iron Age Danes.

**Conclusion:**

The present work provides further evidence that retrieval of ancient human DNA is a possible task provided adequate precautions are taken and well-considered sampling is applied.

## Introduction

Early attempts to retrieve DNA from ancient sources involved molecular cloning [1]–[3], however, with the invention of the PCR technique [4] sensitivity was dramatically improved and soon a sense that “everything” was possible led to numerous reports on DNA sequences from species thousands or even millions of years old (for review see [5]). Eventually it became clear that many of these early results were in fact due to contaminating modern DNA. This was especially true for results on ancient humans because of the abundance of modern human DNA [5]–[7]. In the past few years several studies have taken previous mistakes into account and it has been shown that when strict measures are observed, the recovery of authentic ancient human DNA may be possible [8]–[16]. Recently researchers have pointed out that the generally accepted laboratory “rules” (see e.g. [17]), which are well suited to avoid or account for laboratory derived contamination, may not be sufficient to detect more worrisome contamination added to the material during excavation and subsequent handling (pre-laboratory contamination) [18]–[20]. Furthermore, Sampietro at al. 2006 [19] showed that modern contaminating sequences after about 10 years of storage may show the same amount of damage as is seen in ancient DNA (aDNA), so that “damage patterns” no longer can be argued as an authentication criterion.

We are currently analyzing aDNA from human remains from the Danish and Greenland past to shed light on population heterogeneity, population affinity and family relationships. We have obtained mitochondrial DNA (mtDNA) sequence results on subjects from an early Christian cemetery (AD 1,000–1,250), two Roman Iron Age settlements (AD 0–400) and from Greenland Inuit (AD ∼1,450) 14, 16, 21. In the present work we had the opportunity to obtain samples for aDNA analysis from ten Viking Age [22] subjects that at the time of sampling were untouched by humans for 1,000 years.

## Materials and Methods

### Archaeological site and human remains

**Figure 1:**
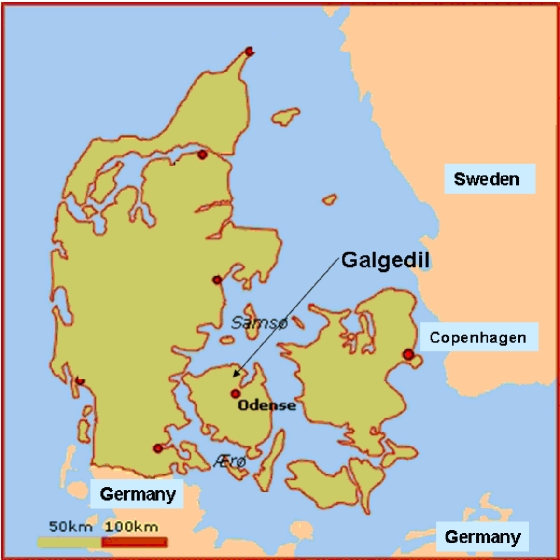
Map of Denmark. The arrow indicates the location of Galgedil in the island of Funen.

**Figure 2:**
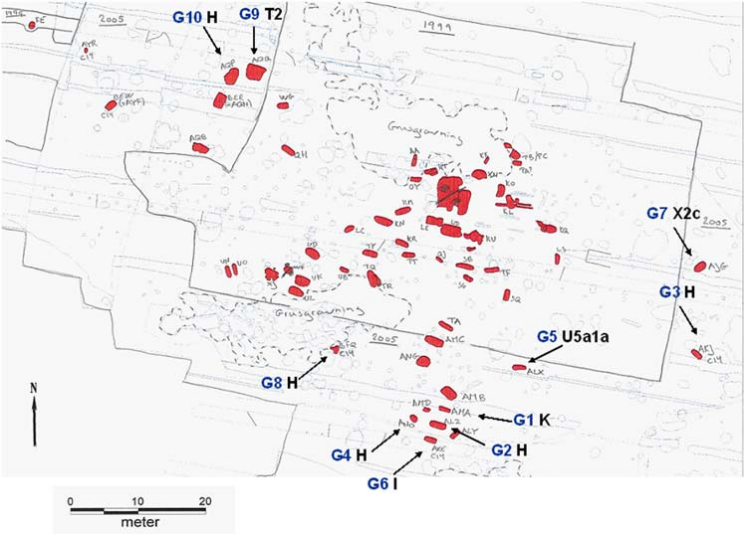
Map of the burials at Galgedil. Galgedil is a large Danish burial ground from the early Viking period. The area in the inner box was excavated from 1999–2002, whereas the outer area was excavated in 2005. The red squares show the graves. The ten subjects analyzed in this work are from the 2005 excavation; subject ID (blue) and mtDNA haplotypes (black) are indicated with arrows. The two areas surrounded by a dotted line have been used as sand pits, which unintentionally may have lead to destruction or removal of graves.

The subjects in the present study are from a non-Christian burial site, Galgedil (AD 700–1100) in the island of Funen (Figs. 1 and 2), which contains at least 59 inhumations and two cremation burials (site excavated in 1988, 1999–2002 and 2005). We obtained samples for aDNA analysis for the present study during the last excavation from subjects who were C^14^ dated to about AD 1,000 (Kirsten Prangsgaard, personal communication).

**Figure 3:**
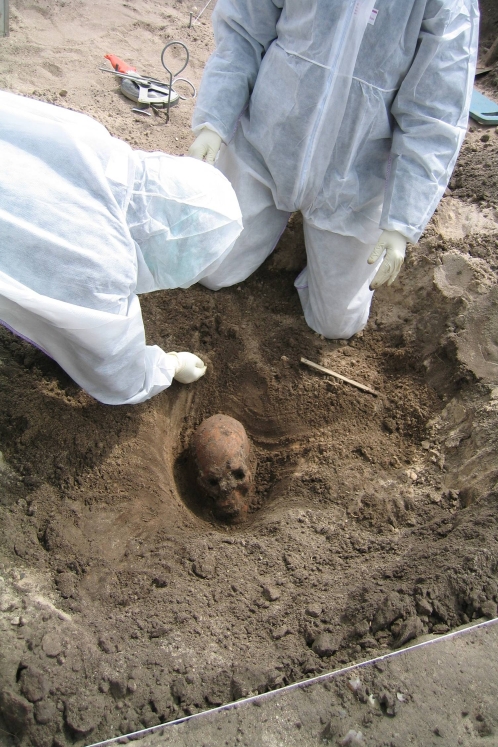
Sampling of teeth for aDNA analysis. The last layer of soil was removed and two teeth extracted while wearing full body suit, hairnet, gloves, shoe covers, and face masks. The teeth were placed in sealed sterile tubes and transported to the aDNA-lab.

The exhumation of ten subjects was performed in a way as to prevent contamination with modern human DNA. Laboratory staff was present during the exhumations and removed the last layer of soil from the skulls and extracted two teeth (preferably premolars or canines) from the jaw wearing full body suit, shoe covers, hairnet, filter-containing facemask, and gloves while being the only persons near the subject (Fig. 3). The sampled teeth were immediately transferred to sealed sterile tubes and transported to the laboratory. Soil samples were taken in close proximity to subjects G1–G3 (see below). The remaining exhumation of each individual was carried out using standard archaeological procedures. Due to the sandy soil the skeletal remains were not cleaned by washing but only brushed. Sexing and aging (performed by Pia Bennike) were performed at the Laboratory of Biological Anthropology, Institute of Forensic Medicine, University of Copenhagen, using a number of anthropological standard criteria when possible [23]–[27] . After the archaeological and anthropological manipulation a third “handled” tooth was sampled from eight of the ten subjects by a researcher from the Research Laboratory using disposable gloves and face mask, and placed in a sealed sterile tube.

The only criteria for including subjects into the present study were that they were adults with at least 3 intact permanent teeth (with closed apex) still sitting firmly in the alveolus and that the last layer of soil was untouched by archaeologists until the above mentioned sampling precautions could be taken. The population sample thus selected comprised three males (G2, G6, G9), four females (G1, G7, G8, G10) and three individuals whose sex could not be determined with certainty but who were tentatively sexed as one female (G3) and two males (G4, G5). For further details see Table 1.

**Table 1:** Nucleotide substitutions and mtDNA haplogroup assignments for Viking remains from Galgedil, AD 1,000

Subject	Age (yrs.) and sex	Coding region	HVR-1 region nt 16064–16405	Haplogroup	Templates^1^	Occurrence among 15,854 individuals from extant populations of Europe and Near East
**G1**, Grave AMA	50+, ♀	7028T, 12308G	16126C, 16224C, 16311C, 16320T	K	8,160	No exact match in European database nor in haplogroup K database [58], related haplotypes exist with either 16126 or 16320 transition.
**G2**, Grave ALZ	45+, ♂	7028C	16278T	H	13,280	Common and widely spread haplotype throughout Europe, 0.16%.
**G3**, Grave AKJ	40–50, ♀?	7028C	16093C, 16212G, 16222T, 16255A	H	60,360	No exact match in the database. Related haplotypes found in Croatian Island population and a Cornish sample.
**G4**, Grave ANO	35–45, ♂?	7028C	16213A	H	19,600	Rare but widely spread haplotype throughout Europe, 0.05%.
**G5**, Grave ALX	30–40, ♂?	7028T, 12308G	16256T, 16270T, 16399G	U5a1a	67,960	Common haplotype throughout Europe, 0.92%.
**G6**, Grave AXE	50+, ♂	10034C	16129A, 16223T, 16391A	I	5,760	Common haplotype throughout Europe, 0.72%. Root haplotype of Hg I.
**G7**, Grave AJG	20–30, ♀	7028T, 14470C, 8705C	16189C, 16223T, 16255A, 16278T	X2c	18,200	Rare but widely spread haplotype throughout Europe, 0.11%.
**G8**, Grave BFQ	50+, ♀	7028C	16174T	H	4,720	Rare type observed in Eastern Europe, 0.02%.
**G9**, Grave AQQ	25–35, ♂	7028T, 15607G	16126C, 16294T, 16296T, 16304C	T2	375,120	Common haplotype throughout Europe, 1.71%.
**G10**, Grave AQP	45+, ♀	7028C	16172C, 16304C	H	279,320	Rare but widely spread haplotype throughout Europe, 0.03%.

1 Templates, number of DNA molecules in extracts from either tooth #1 or #2.

The last column compares the haplotypes observed with 15,854 entries in a private world database maintained by one of us (TK). Haplotypes occurring at >0.5% in the database are considered as “common”, following the frequency criteria used in the Coble et al. 2004 study [59]. It should be noted that rare haplotypes that occur across wide geographic space might occur due to parallel mutations in the HVR-1 region.

### Ancient DNA-work

To ensure the highest possible reliability of the present work some of the most general and widely accepted guidelines for aDNA work that have been suggested (see e.g. [17], [18]) were followed and additional elements were added. This has previously been described in detail in [16]. The following is a summary of the various steps:

### Clean-Laboratory

All pre-PCR manipulation and cutting of teeth, extraction of DNA and mixing of reactions for PCR was performed in a “Clean-Laboratory” dedicated solely to aDNA work.

### Chemicals, reagents and PCR-and centrifuge tubes

All chemicals and reagents were of analytical grade or the highest purity available unless otherwise stated. PCR tubes and micro centrifuge tubes for extracts and primers were free of human DNA as guaranteed by the manufacturer (“PCR-Clean and “Biopure” tubes, Eppendorf, Hamburg, Germany).

### Extraction of DNA, PCR, cloning and sequencing

mtDNA was isolated from pulp material. One tooth was prepared and extracted at a time. The tooth was wiped with diluted commercial bleach containing 0.2% hypochlorite, cleaned with water and the surface sealed with lacquer. After cutting the tooth pulp was drilled and DNA was extracted using the dialysis approach of [9]. Dissolved DNA was collected on a 30 kDa filter (Microcon, Millipore, Glostrup, Denmark) and purified using the QIAamp DNA mini kit (Qiagen, Venlo, The Netherlands) and eluted with 2×100 µl de-ionized, filtered and autoclaved water. The extract was stored at –20°C in 25 µl aliquots. Independent extraction and analysis of DNA from a second tooth and a third “handled” tooth was performed by different researchers at intervals of several weeks. Extraction of human DNA from soil was performed according to [28] . DNA was amplified and sequenced over the mitochondrial Hyper Variable Region 1 (HVR-1) between nucleotides (nt) 16064–16405 (the revised Cambridge Reference Sequences, rCRS [29]) using 4 overlapping fragments (A, B, C, D) of between 135–141 bp. An 113bp segment containing nucleotide position 7028 which is informative of haplogroup H was also amplified for all samples. In addition, all non-H samples were amplified over coding region segments containing haplogroup specific substitutions (Table S1). Two to 7.5 µl extract were used per PCR reaction (25 µl, 40 cycles) depending on the DNA concentration. Positive PCR reactions were cloned and an average of 8 clones was sequenced for each PCR reaction. The sequences were aligned against the rCRS sequence, analyzed for post-mortem damage induced miscoding lesions and the consensus sequence was determined.

### Haplogroup analysis and statistical analyses

Haplogroup (Hg) affiliations were assigned following the established rules and definitions [30], [31] and population affinities by haplotype/group frequencies were determined by comparison with published data for extant populations of Europe and Near East using a private mtDNA database maintained by one of us (TK). Haplotype consistency was analyzed by construction of the median-joining network [32] relating the HVR-1 sequences and coding region substitutions. Statistical comparison of the Galgedil population sample with previously analyzed Danish Viking and Roman Iron-Age population samples (Kongemarken, Skovgaarde and Bøgebjerggård, respectively) was performed using permutation based Monte Carlo simulations of the HVR-1 sequence data using 1,000 simulations per comparison (kindly performed by Hans Siegismund, Section of Evolutionary Biology, Institute of Biology, University of Copenhagen) [33].

### Real time quantitative PCR analyses

The DNA content of the DNA extracts was quantified by quantitative real time PCR (qPCR) using the ABI Prism 7000 Sequence Detection System and the Power SYBR Green PCR Master Mix (Applied BioSystems, Foster City, CA, USA). Primers for a 143-bp mtDNA segment from nt 8294 to 8436 were used as described by Andréasson et al. (2002) [34]. A mtDNA standard was produced as described previously [14] by cloning a 302-bp segment containing the aforementioned segment using the pGEM Easy Vector system (Promega, Madison, WI, USA). The standard was determined in 5 dilutions: 100, 1,000, 10,000, 100,000 and 1,000,000 copies per reaction. The DNA extracts were determined in two dilutions, usually 2x and 10x, to estimate the effect of possible inhibitors in the extracts. Further, to help suppress inhibition each PCR reaction contained 25 µg bovine albumin (Ultrapure BSA, Ambion, Austin, TX, USA). Five µl DNA extract was added to triple qPCR reactions (25 µl). The final results reflect the approximate copy number of the 143-bp mitochondrial fragment in 200 µl extract.

### DNA from staff

DNA from archaeologists and anthropologists (A1–A6) were obtained using EasiCollect for buccal cell collection (Whatman, Maidstone, Kent, UK). The extraction of DNA was carried out in accordance with the manufactures instructions. Two overlapping segments of mtDNA HVR-1 were amplified using primer pairs L16063/H16228 and L16225/H16406 (Table S1), cloned and sequenced using the standard procedure described above. Based on the substitutions identified in HVR-1, appropriate diagnostic sites in the coding region were analyzed to confirm haplotypes.

DNA from the remaining staffs (R1–R5, A7 and A8) was sequenced over the HVR-1 region at the Department of Forensic Genetics, Institute of Forensic Medicine, University of Copenhagen using standard procedures [35]. All samples were voluntarily taken by the staffs and submitted with written consent. The Regional Board of Ethics has approved the usage of DNA from staffs in the present work.

### Control Inuit DNA

To test the overall reliability of the laboratory procedures used (reagents, extraction method, cleaning methods, possible background contamination) a tooth from an Inuit skull from a burial site in Greenland (approximately 500 YBP) was extracted, PCR amplified, cloned and sequenced over the A-segment [16], [21].

## Results

**Figure 4:**
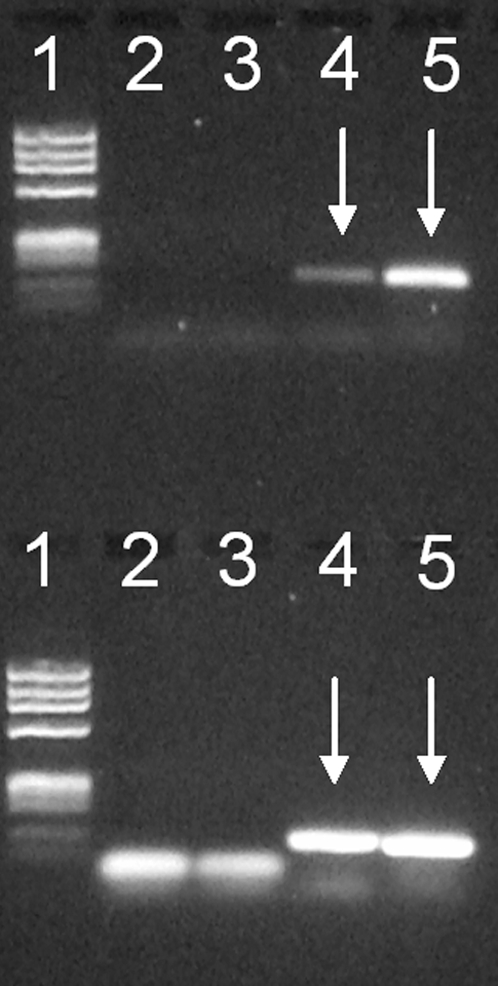
Mini gel patterns of PCR amplified mtDNA. PCR amplification of a fragment spanning the mtDNA HVR-1 region from nt 16307–16405 (D-fragment) and a fragment (H-fragment) harbouring nt 7028 (nt 6987–7047) using extract from subject G2. Upper row, lanes 1–5, D-fragment: 1, size standard (ΦX174 RF DNA HeaIII-digested); 2, PCR blank; 3, mock extraction; 4, extract; 5, positive DNA control. Lower row, lanes 1–5, H-fragment, same order as for D-fragment. Arrows indicate mtDNA bands.

**Figure 5:**
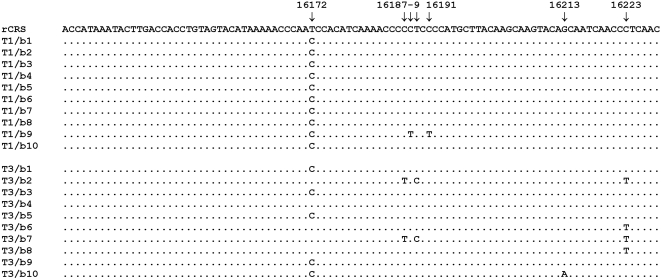
Alignment of cloned mtDNA sequences (B-segment: nt 16132–16228) from two teeth from subject G10 from Galgedil. rCRS, revised Cambridge reference sequence [29]; T1 and T3, tooth #1 and #3 from individual G10. Tooth #1 was untouched by humans prior to sampling while tooth #3 was sampled after archeological and anthropological manipulation. b1-10, clones 1-10 of the B-segment. The transition at nt 16172 was observed with DNA extracts from all three teeth from subject G10 (tooth #2 not shown) and is presumed to be a true substitution in the authentic DNA. Five of the clones from tooth #3 show diverging sequences (b2, b4, b6, b7 and b8) which lack the transition at nt 16172. Four of the diverging sequences harbor a transition at nt 16223 and two of these (b2 and b7) show additional transitions at nt's16187 and 16189. The fifth diverging sequence, b4, is rCRS and lacks any substitutions. The diverging sequences are considered to represent exogenous DNA, but they do not match any of the staffs (Table 2). The substitutions at nt's 16188, 16191 and 16213 are presumed to be the result of scattered post-mortem miscoding lesions, which is a characteristic finding for ancient DNA [38].

We have assessed mtDNA variation in ten Viking subjects from Galgedil, ca. AD 1,000, in the northern part of Funen, Denmark. All subjects were untouched by humans at the time of sampling and additional precautions were taken to minimize the risk of pre-laboratory contamination. mtDNA was extracted independently from two teeth, PCR amplified, cloned and sequenced. No bands were observed for PCR blanks or mock extractions (Fig. 4). Reproducible sequence results were obtained from the two teeth from each subject and alignment of multiple clones showed no evidence of contamination (Tables 1 and S2 and Fig. 5). It should be noted, however, that there is a match between G9 and R2 (Table 2) who shares a frequent haplotype in haplogroup T2. R2 has not been involved in the sampling of teeth or the analysis of the aDNA and a uniform contamination of these two teeth and a third tooth mentioned below with DNA from R2 is highly unlikely. Scattered substitutions, which usually accumulate in aDNA as a result of post-mortem spontaneous damage reactions were observed [36]–[41], while consistent substitutions shared by all clones for a given segment were believed to represent the authentic ancient sequence. Furthermore, a test of the laboratory procedures during the study using an ancient Inuit tooth showed the typical Inuit substitution at nt 16111 in the A-segment for all clones [16], [42]. These results allowed the unequivocal assignment of a specific haplotype to each of the ten subjects (Table 1).

**Table 2:** Nucleotide substitutions and mtDNA haplogroup assignments for staffs involved in the excavation and analyzes of Viking remains from Galgedil

Subject	HVR-1 region nt 16064–16405	Haplogroup
**R1**	16298G	V
**R2**	16126C, 16294T, 16296T, 16304C	T2
**R3**	16192T, 16239T, 16256T, 16270T	U5
**R4**	16129A, 16293G, 16311C	H11
**R5**	CRS	H
**A1**	16189C	H
**A2**	16162G	H1a
**A3**	16051G, 16162G	H1a
**A4**	16294T, 16296T, 16304C	T2
**A5**	16069T, 16093C, 16126C, 16145A, 16172C, 16222T, 16261T	J1b1
**A6**	16189C, 16192T, 16256T, 16270T, 16294T	U5a1
**A7**	16304C	Not determined
**A8**	16362T, 16390A	Not determined

R1–R5, staffs at the Research Laboratory; A1–A8, archaeological and anthropological staffs.

In contrast with the above results, mtDNA extracted from a third “handled” tooth, which was obtained from the skulls after standard archaeological and anthropological manipulations, showed evidence of contamination with extraneous human DNA; diverging sequences were observed among the authentic sequences in extracts from four out of eight teeth (Fig. 5 and Table S2). Surprisingly, none of these diverging sequences matched the staffs involved in the handling steps, and the origin of these sequences remains unclear (Table 2). The possibility of old human contamination was considered. However, analysis of soil samples taken in close proximity to subjects G1, G2 and G3 at the time of excavation showed no evidence of human DNA; also it is unlikely that an old contamination would only affect the “handled” teeth. The absence of staff related sequences may reflect awareness among the archaeological staff about the risk of contamination and the fact that the remains were only brushed (due to the sandy soil) and not washed as is normally the case during the archaeological rinsing of skeletal remains.

Quantitative PCR analysis of the DNA extracts showed DNA template levels between 4,720–375,120 molecules in 200 µl extract (Table 1). There was no consistent indication that the “handled” teeth contained more DNA than the “untouched” teeth (results not shown).

**Figure 6:**
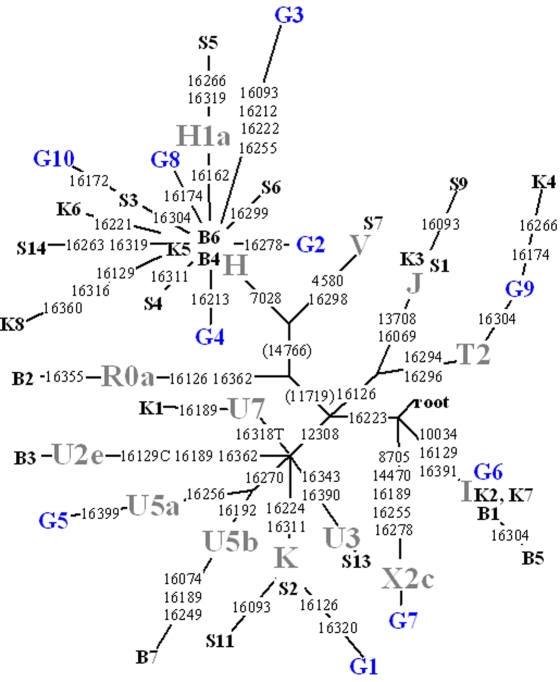
Median-joining network of ancient Danes. Median-joining network relating 36 HVR-1 sequences (nt 16064–16405) of Danish Viking (Galgedil (10 subjects) and Kongemarken (8 subjects)) and Roman Iron Age (Bøgebjerggård (7 subjects) and Skovgaarde (11 subjects)) samples genotyped for mtDNA haplogroup defining coding region substitutions. Variable sites are shown along the branches of the network. Substitutions at nucleotide positions 11719 and 14766 (shown in parentheses) were inferred from the haplogroup trees drawn using completely sequenced mtDNA genomes [31], [60], [61]. Reticulation between haplogroups, e.g. R0a vs JT (16126 parallelism) and U1 vs U7 (16189 parallelism) were solved manually considering phylogenetic analyses based on complete sequence data. L3 is used as the root. Sample codes correspond to Tables 1 and S3; haplogroup labels are shown in grey font.

To test for phylogenetic consistency, the haplotypes observed for the Viking subjects were analyzed by the construction of the most parsimonious tree through the median-joining network approach (Fig. 6). No unusual substitution motifs were observed. Sequences from two Iron Age villages, Bøgebjerggård and Skovgaarde [16], and an early Christian cemetery, Kongemarken [21] were added to the tree, to see how haplotypes from the various sites fit with each other (Table S3). As shown in Fig. 6 there was no indication that any of the sites form a separate cluster. Furthermore, statistical comparison of the Galgedil population sample with the population samples from Kongemarken, Skovgaarde and Bøgebjergård showed that the distribution of haplotypes does not deviate significantly (K_ST_ = 0 and P = 0.561, 0.373 and 0,624, respectively [33]). Several haplogroups that are rare amongst present day Scandinavians were observed in the Galgedil population (three subgroups of Hg H, one subtype of Hg K and one subgroup of Hg X (Table 1)).

## Discussion

### Authenticity of results

Providing convincing evidence for the successful retrieval of aDNA-especially aDNA of human origin-is a demanding task, and even results that were obtained using extensive precautions have later been disputed and claimed to be due to contaminating modern DNA [18], [43]–[46]. The fundamental problem with analysis of ancient human DNA is the abundance of modern human DNA in most contemporary settings. Sampietro et al. (2006) [19] recently showed that archaeological manipulation of human remains is a major source of contamination with modern DNA and Pruvost et al. (2007) [47] showed that once skeletal remains are stored after excavation the authentic DNA seems to degrade rapidly.

In the present work we have circumvented the above two obstacles by taking teeth from Viking remains at the excavation site at the moment the jaw was accessible. We removed the last layer of soil from the skulls and extracted premolars from the jaws wearing protective outfit. The results support the absence of pre-laboratory contamination–in fact any contamination with human DNA, i.e. alignment of multiple cloned sequences showed no evidence of the existence of more than one mtDNA sequence from any given subject, and the sequences did not match with any of the staffs (archaeologists, anthropologist and laboratory staff except for subject G9 which has been accounted for above (see Results)). The spectrum of haplotypes observed for the Viking samples, i.e. ten different haplotypes for ten individuals, further strengthens the reliability of the results. Thus it would be highly unlikely that ten plausible haplotypes all fitting well within the phylogenetic tree would arise from the random combination of short authentic fragments and/or an undetected low background of contaminating DNA in the laboratory environment.

The risk that post-mortem miscoding lesions in the template DNA lead to false conclusions about the true authentic sequence has been debated [5], [17], [37], [48]–[50]. The fewer template molecules being available for the PCR amplification the higher the risk of drawing wrong conclusions. Thus, the risk is considered to be low when the PCR is initiated with several hundred template molecules [49] and as a rule of the thumb reliable DNA sequences may be obtained from a single PCR if more than 1,000 starting molecules are present [5]. In the present work extracts (200 µl) of ancient DNA contained between 4,720 and 375,120 molecules, i.e. 177 ancient templates were present in PCR reactions with the most dilute extract (using 7.5 µl) and thousands of molecules were available with the more concentrated extracts. Given that the present results are all based on multiple clones from PCRs of extracts from at least two (usually three) teeth the likelihood of the ancient sequences presented here being due to artefacts seems very low.

### Rare mtDNA haplogroups

Given the small sample sizes the Viking population sample from Galgedil does not differ significantly from other Viking and Iron Age population samples from the Danish past by the haplogroup frequency distribution, however, it is noted that five of the ten subjects harbour mtDNA haplotypes which have either not been observed or are infrequent in modern Scandinavians (Table 1). In particular the observation of haplotype X2c is interesting (subject G7). Haplogroup X is itself rare (0.9% in Scandinavians [51]) but has a very wide geographic range, and X2c is a rare subgroup of X accounting for only 5% of 175 Hg X samples surveyed in 2003 [52]. A possible European (Viking?) origin of haplotype X2a identified among Native Americans has been suggested [53], [54], but X2a has not been detected in Europe and the present observation of X2c amongst the Vikings does not support this proposal.

Among present day Scandinavians Hg I constitutes <2% [55], [56], however, we have previously observed a markedly higher frequency (10–20%) of Hg I in Danish Iron Age and Viking Age population samples (Table S3) [16], [21]. With the observation of Hg I for subject G6 this trend is also seen for the Viking population sample from Galgedil. Interestingly, Hg I shows a low frequency (1 out of 114 subjects) among other ancient populations in Italy, Spain, Great Britain, and early central European farmers [11], [12], [43], [57].

### Concluding remarks

The present work provides further evidence that retrieval of authentic DNA from ancient humans is indeed a possible undertaking provided adequate precautions are observed. The importance of proper sampling precautions is underscored by the evidence of contaminating sequences in four out of eight teeth that were taken from the ancient subjects after standard manipulation of the skeletal material by archaeologists and anthropologists. A reliable retrieval of authentic DNA opens the way for a valuable use of prehistoric human remains to elucidate the genetic history of past and extant populations.

## Supporting Information

Table S1 Oligonucleotide primers used for DNA-amplification. Number in primer name indicates position of 3′ nucleotide [29].
(0.04 MB DOC)

Table S2 Number of diverging clones among total number of sequenced clones from each of eight. The number (29) of diverging sequences may be higher than stated in the table, since the only way to identify diversion is the absence of defining substitutions as observed in 16 of these sequences or the presence of one or more diverging substitutions (11 C→T, 2 G→A, 2 T→C). C→T and G→A transitions are frequently observed in DNA that has been damaged post mortem [19], [40]. Therefore, it cannot be ruled out that those sequences that harbor only on or the other of these two latter substitutions may be the result of damage derived modifications of older contaminating human DNA or of the authentic ancient DNA.
(0.02 MB DOC)

Table S3 Nucleotide substitutions and mtDNA haplogroup assignments for previously analyzed Danish Iron Age and Viking Age subjects. B1–B7, Bøgebjergård (Iron Age). S1–S14, Skovgaarde (Iron Age). K1–K8, Kongemarken (Viking Age) [16], [21].
(0.06 MB DOC)
