# Supplementary material for: Evidence of Authentic DNA from Danish Viking Age Skeletons Untouched by Humans for 1,000 Years
Source: PLoS One. 2008 May 28;3(5):e2214. doi: 10.1371/journal.pone.0002214 (PMC2386972; doi:10.1371/journal.pone.0002214)
Supplement: Table S1 — Oligonucleotide primers used for DNA-amplification. Number in primer name indicates position of 3′ nucleotide [29]. (0.04 MB DOC) [file pone.0002214.s001.doc]

| Primer pairs | Sequence (5’→3’) | Length of product (bp) | Annealing |
| --- | --- | --- | --- |
| HVR-1 region | | | |
| L16063  H16161 | TTGGGTACCACCCAAGTAT  GATGTGGATTGGGTTTTTA | 135, A-fragment | 50°C |
| L16131  H16228 | CACCATGAATATTGTACGGT  TTGCAGTTGATGTGTGATAG | 136, B-fragment | 50°C |
| L16225  H16325 | AAGTACAGCAATCAACCCTC  CTGTAATGTGCTATGTACGGTA | 141, C-fragment | 50°C |
| L16307  H16406 | TACCCACCCTTAACAGTACA  TATTGATTTCACGGAGGA | 136, D-frament | 50°C |
| Coding region | | | |
| L6987  H7047 | GCATTGTATTAGCAAACTCAT  GCAAATACAGCTCCTATTGA | 100 | 50°C |
| L9998  H12368 | TCTAATGATGAGGGTCTTACTCTTTT  cGAAGTTTATTACTCTTTTTTGAATGTT | 96 | 56°C |
| L12254  H12368 | ATCCCCCCATGTCTAACAAC  GGGGAATTAGGGAAGTCAGG | 153 | 56°C |
| L14459  H15514 | CTCCTCAATAGCCATCG  GGGAGGTTATATGGGTTTAA | 91 | 50°C |
| L8651  H8713 | TATCTCATCAACAACCGACT  GATCAGGTTCGTCCTTTAGT | 101 | 50°C |
