# Supplementary material for: Evidence of Authentic DNA from Danish Viking Age Skeletons Untouched by Humans for 1,000 Years
Source: PLoS One. 2008 May 28;3(5):e2214. doi: 10.1371/journal.pone.0002214 (PMC2386972; doi:10.1371/journal.pone.0002214)
Supplement: Table S2 — Number of diverging clones among total number of sequenced clones from each of eight. The number (29) of diverging sequences may be higher than stated in the table, since the only way to identify diversion is the absence of defining substitutions as observed in 16 of these sequences or the presence of one or more diverging substitutions (11 C→T, 2 G→A, 2 T→C). C→T and G→A transitions are frequently observed in DNA that has been damaged post mortem [19], [40]. Therefore, it cannot be ruled out that those sequences that harbor only on or the other of these two latter substitutions may be the result of damage derived modifications of older contaminating human DNA or of the authentic ancient DNA. (0.02 MB DOC) [file pone.0002214.s002.doc]

| Subject | G1 | G2 | G4 | G5 | G6 | G8 | G9 | G10 |
| --- | --- | --- | --- | --- | --- | --- | --- | --- |
| Diverging sequences/  number of sequenced clones | 0/38 | 0/40 | 8/38 | 0/29 | 10/35 | 0/37 | 3/32 | 8/41 |
