# Supplementary material for: Evidence of Authentic DNA from Danish Viking Age Skeletons Untouched by Humans for 1,000 Years
Source: PLoS One. 2008 May 28;3(5):e2214. doi: 10.1371/journal.pone.0002214 (PMC2386972; doi:10.1371/journal.pone.0002214)
Supplement: Table S3 — Nucleotide substitutions and mtDNA haplogroup assignments for previously analyzed Danish Iron Age and Viking Age subjects. B1–B7, Bøgebjergård (Iron Age). S1–S14, Skovgaarde (Iron Age). K1–K8, Kongemarken (Viking Age) [16], [21]. (0.06 MB DOC) [file pone.0002214.s003.doc]

| Subject (grave number, sex, age (years)) | Coding sequence | HVR-1 region 16055-16405 | Haplogroup |
| --- | --- | --- | --- |
| **B1,** E**,** ♀, 35-45 | 7028T, 10034C | 16129A, 16223T, 16391A | I |
| **B2,** 4, ♂, 30-40 | 7028T | 16126C, 16355T, 16362C | R0a |
| **B3,** 6, ♂, 40 | 7028T, 12308G | 16129C, 16183C, 16189C, 16362C | U2e |
| **B4,** 7**,** ♂, 25-30 | 7028C | CRS | H |
| **B5,** B, ♀, 25-30 | 7028T, 10034C | 16129A, 16223T, 16304C, 16391A | I |
| **B6,** A, ♂, 18-19 | 7028C | CRS | H |
| **B7,** marts 2000, ♂, 35-45 | 7028T, 12308G | 16074G, 16189C, 16192T, 16249C, 16270T | U5b1 |
| **S1,** 207, ?, 14-16 | 7028T, 13708A | 16069T, 16126C | J |
| **S2,** 5, ♂, 50 | 7028T, 12308G | 16224C, 16311C | K |
| **S3,** 4, ♀, 40-50 | 7028C | 16304C | H1 |
| **S4,** 208, ?, 14-16 | 7028C | 16311C | H |
| **S5,** 7, ♀, 30-35 | 7028C | 16162G, 16266T, 16319A | H |
| **S6,** 1943, ♀, 25-30 | 7028C | 16299G | H |
| **S7,** 209, ♀, 20 | 7028T, 4580A | 16298C | V |
| **S8,** 400, ♀, 25-30 | No results | No results | - |
| **S9,** 204, ♀, 55 | 7028T, 13708A | 16069T, 16093C, 16126C | J |
| **S10,** 202, ♂?, 18-20 | No results | No results | - |
| **S11,** 205, ♀?, 25 | 7028T, 12308G | 16093C, 16224C, 16311C, | K |
| **S12,** 9, ♀, 55-58 | No results | No results | - |
| **S13,** 8, ♀, 20-30 | 7028T, 12308G | 16343G, 16390A | U3a |
| **S14,** 201, ♀, 45 | 7028C | 16263C, 16319A | H |
| **K1,** A396, ♂, 45+ | 7028T, 12308G | 16189C, 16318T | U7 |
| **K2,** A367, ♀, 25-30 | 7028T | 16129A, 16223T, 16391A | I |
| **K3,** A368, ♀, 45+ | 7028T | 16069T, 16126C | J |
| **K4,** A362, ♀, 45+ | 7028T | 16126C, 16174T, 16266T, 16294T, 16304C | T2 |
| **K5,** A211, ♀, 30-40 | 7028C | CRS | H |
| **K6,** A386, ♂, 25-40 | 7028C | 16221T | H |
| **K7,** A363, ♀, 18 | 7028T | 16129A, 16223T, 16391A | I |
| **K8,** A395 ,♀?, 15 | 7028C | 16129A, 16316G, 16360T | H |
